# Home-Based Virtual Reality Exergame Program after Stroke Rehabilitation for Patients with Stroke: A Study Protocol for a Multicenter, Randomized Controlled Trial

**DOI:** 10.3390/life13122256

**Published:** 2023-11-26

**Authors:** Dongheon Kang, Jiyoung Park, Seon-Deok Eun

**Affiliations:** Department of Healthcare and Public Health Research, National Rehabilitation Center Ministry of Health and Welfare, Seoul 01022, Republic of Korea; jakekang@korea.kr

**Keywords:** stroke, virtual reality, rehabilitation, exergame, exercise

## Abstract

It is essential for stroke patients to maintain their therapy even after discharging inpatient rehabilitation. This is because recovery is an ongoing process that requires consistent effort. Virtual reality exergame training (VRET) is becoming widely used in stroke rehabilitation to improve physical, social, and psychological outcomes. Home-based VRET may be a more convenient and accessible option for stroke rehabilitation. This study will aim to determine the effectiveness of home-based VRET for patients with stroke who have been discharged from the hospital. This trial will randomly assign 120 participants to 8 weeks of either a VRET (intervention group) or daily life (control group). The study will measure cardiopulmonary endurance, muscular strength, functional capacity, gait, activities of daily living, and quality of life. Our main objective is to determine whether it is safe for patients to undergo VRET at home after they have been discharged from the hospital with a doctor’s note. Additionally, we aim to examine whether stroke patients are capable of exercising at home after being discharged from the hospital. This study’s outcome could pave the way for developing more comprehensive exercise protocols for stroke patients. Our findings will provide valuable insights into the efficacy of VRET as a therapeutic tool for stroke patients.

## 1. Introduction

In recent decades, there has been a significant increase in the incidence of stroke, making it a leading cause of long-term disability and death on a global scale [1,2]. Presently, stroke ranks as the second and third most significant cause of mortality on a global and South Korean level, respectively [3,4]. South Korea alone witnesses approximately 105,000 individuals experiencing either their first or recurrent stroke every year [5].

Many stroke patients spend about 10 weeks in the hospital recovering from their stroke, and they are discharged to the community before they can fully recover. During this period, the central nervous system experiences heightened neuroplasticity, allowing for significant functional improvement [6,7]. Early intervention following a stroke has contributed to a decrease in the mortality rate and an increase in the survival rate. However, managing stroke patients after their hospital discharge has become a crucial concern for ensuring secondary prevention and a healthy life for these patients [3,8,9,10]. The effectiveness of therapeutic outcomes is heavily dependent on treatment intensity, with highly repetitive, task-oriented, intensive, and task-specific therapies proving to be the most effective [11,12]. To achieve the best possible recovery, stroke patients require continuous and intensive treatment [13].

Typically, post-discharge patients are offered outpatient therapy. However, it is not always feasible for stroke patients to attend outpatient treatment due to various factors, such as distance, transportation issues, and adverse weather conditions, affecting their ability to travel from home to a rehabilitation facility [13]. Additionally, these programs often have waitlists, and each patient is granted only a limited number of sessions.

Home-based training can play a pivotal role in providing improved access to rehabilitation. This approach allows patients to enhance or extend their training, potentially leading to better outcomes [13]. According to rehabilitation guidelines, individuals are encouraged to engage in self-directed training with daily objectives [14,15].

Non-immersive virtual reality training (VRT) involves the use of a computer-based software program that tracks the user’s movements, enabling them to interact with a game or activity displayed on a screen. It offers a repeatable, convenient, and enjoyable experience for patient recovery after a stroke. Furthermore, it comes with no restrictions on usage [16,17]. Specifically, non-immersive virtual reality exergame training (VRET) refers to video games that require whole-body movements, enabling real-time interaction [18]. Several studies have shown that VRET can improve various aspects of physical fitness, such as cardiorespiratory fitness, muscle strength, physical function, and mobility, after a stroke. It has been found to be equally or more effective than traditional therapy [16,19,20,21,22,23]. VRET also offers an alternative to traditional repetitive physical exercises, creating engaging and multisensory game environments that involve whole-body movements for interaction [24]. This breaks the monotony of traditional therapy and makes exercise training more enjoyable. Gamified approaches and immersive scenarios can motivate stroke patients to engage in more physical and rehabilitation exercises after hospital discharge [25]. This approach can reduce the time needed for staff intervention while motivating patients to engage in high-energy movements. Therefore, VRET is a promising complement or alternative to traditional rehabilitation programs and can significantly impact the lives of patients after their hospital discharge.

Several studies have explored the potential application of home-based VRET for improving physical fitness and physical performance recovery following a stroke, demonstrating the feasibility of home-based VRET systems for continuous rehabilitation at home [26,27,28,29]. However, after discharge, patients with stroke participating in home-based VRET programs require established strategies by which to evaluate their physical fitness and functional limitations and optimize their physical fitness and functional improvements. Previous studies have shown that participants were delighted with home-based VRET, although actual program implementation can vary considerably [27,30]. Barriers to using home-based VRET also include the fact that many people have insufficient technological experience and technical issues are challenging [31].

Home-based VRET shows promise as a supplementary or alternative rehabilitation program for stroke survivors, potentially having a significant impact on their lives [13]. The feasibility of utilizing home-based VRET to improve post-stroke physical fitness after hospital discharge has been explored [27,30,32,33]. Thus, home-based VRET systems show promise for home-based rehabilitation.

Exergame-based VRT offers an engaging and multisensory approach to physical exercise that eliminates the barriers of monotony. By immersing users in interactive environments and performing full-body exercises, VRT provides a fun and effective way to remain active [24]. Patients with a stroke are more motivated to engage in physical and rehabilitative exercise practices using gamified approaches and immersive scenarios [13]. Therefore, playing exergames at home reduces intervention time, encourages high-energy movements, and boosts motivation for patients compared with hospital settings.

According to the American Heart Association, survivors of stroke are advised to improve their physical fitness, including cardiopulmonary endurance, muscular strength, and functional capacity [34]. However, many stroke patients living in the community after discharge from rehabilitation tend to participate in activities that lack physical activity or are not significantly conducive to physical recovery [35,36,37]. Additionally, the exercise participation of patients after stroke at home is limited, and safer exercise methods are required [38,39]. One of these new methods is home-based VRET with a doctor’s note to ensure the safety of patients after stroke while performing exercise training.

Stroke patients who have completed rehabilitation in the hospital and returned to the community should be screened for the effectiveness of a VRET program that takes into account their physical ability based on their condition and their doctor’s note. However, there are no studies in South Korea that analyze whether stroke patients return to the community after being discharged from the hospital and participate in VRET safely at home with a doctor’s note. Thus, this research team, which consists of rehabilitation medicine experts and exercise experts, aims to investigate the effects of VRET at home for patients with stroke after being discharged from the hospital with a doctor’s note.

## 2. Materials and Methods

### 2.1. Study Design

This trial will be a multicenter, randomized, double-blind, parallel-group trial including patients who have experienced stroke for the first time. Participants will be randomly allocated to the control or experimental group in a 1:1 ratio, with instructions not to disclose their group assignment to the assessors for future evaluations. The study will be performed by teams at four hospitals in the Republic of Korea: (1) National Rehabilitation Hospital, (2) Korea University Anam Hospital, (3) National Health Insurance Service Ilsan Hospital, and (4) Bucheon SM Hospital.

The potential participants will be recruited from the hospitals’ stroke rehabilitation programs for both inpatients and outpatients. An investigator conducts the evaluation with the consent of the study participants during the study and cannot confirm the assignment of the participants.

**Figure 1:**
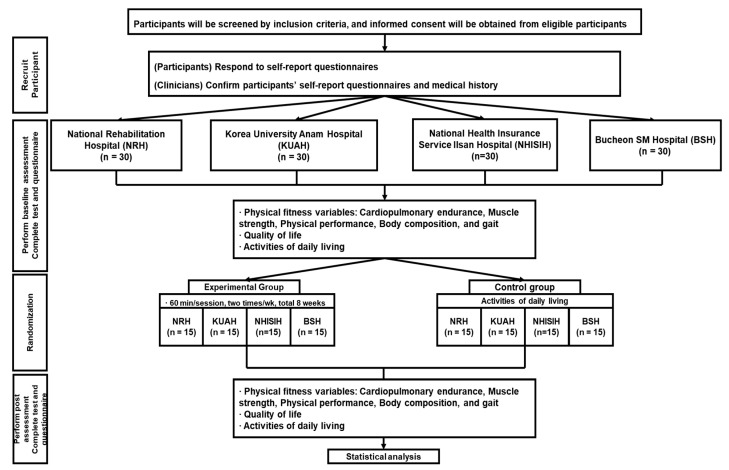
Study flowchart. NRH, National Rehabilitation Hospital; KUAH, Korea University Anam Hospital; NHISIH, National Health Insurance Service Ilsan Hospital; BSH, Bucheon SM Hospital.

The study protocol will be initiated and managed by the National Rehabilitation Hospital. This study was approved by the National Rehabilitation Hospital Institutional Review Board (NRC202105043). The study protocol was registered and assigned KCT0007521 (first registration 19/07/2022). The protocol conforms to the SPIRIT [40]. The study’s flowchart is presented in Figure 1.

### 2.2. Participants

Individuals who have experienced a stroke may be considered for the study if they meet the following criteria: (1) they have had an ischemic or hemorrhagic stroke; (2) they have been hospitalized for a stroke and have returned to the community; and (3) they willingly agree to enroll in the study. 

Patients will be excluded from the trial if they (1) are currently hospitalized; (2) are unable to engage in physical activity owing to neurological disorders (except stroke), orthopedic issues in their lower limbs, or heart or lung problems; or (3) are incapable of completing the assigned challenge on the basis of evaluator’s assessment.

### 2.3. Randomization

After the participants have signed the informed consent form, the supervisor of each team will refer eligible participants for the study. Afterward, a statistician, independent of the study team, will perform random assignments. The participants within each institution will be stratified into two subgroups based on a stratified block randomization method with blocks of size two within each stratum. The assignment ratio of the experimental and control groups will remain at 1:1.

The random assignment list will be created through SPSS version 23.0 (IBM Corp. located in Armonk, NY, USA). The statistician in charge of random assignment will randomly select the block size and seed number. Subsequently, the random assignments will be placed in opaque envelopes for each participant and handed to the coordinator at the beginning of the intervention. 

### 2.4. Randomization and Blinding

This clinical trial will maintain a double-blind protocol, ensuring both participants and evaluators remain unaware of group assignments. Furthermore, instructors administering intervention will also be blinded throughout the study. Instructors administering intervention will also be blinded throughout the study to mitigate potential bias and ensure the objectivity of the results, thereby enhancing the validity of the research findings. However, due to the nature of this trial, the investigator responsible for performing the study will not be blinded to the assigned groups. 

### 2.5. Protocol

The research team will assess whether potential participants meet the inclusion or exclusion criteria on their initial visit. Additionally, the researcher will clarify the study’s specifics, secure informed consent through the provision of consent forms, and brief the participants on the study. Written informed consent will also be collected. Following this, participants will complete a self-report questionnaire and submit it to a clinician for a health assessment and review of the questionnaire’s content. Before random assignment, qualified assessors will assess each participant’s outcome variables. 

### 2.6. Program

Table 1 displays the details of the intervention program, consisting of 60 min sessions held twice a week for a total duration of 8 weeks.

**Figure 2:**
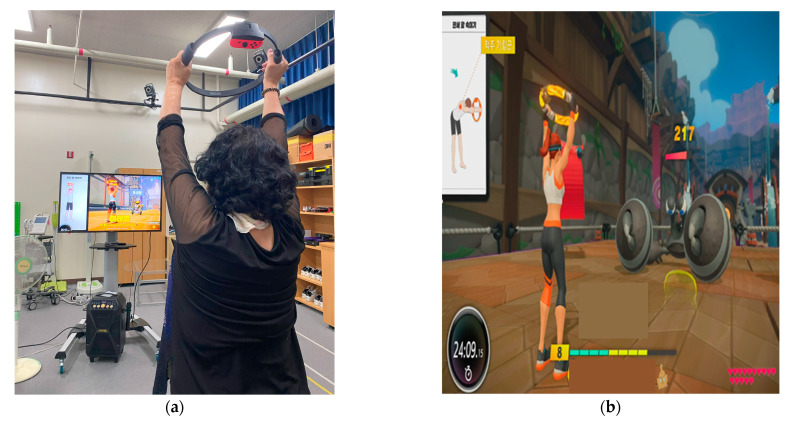
Experimental intervention for virtual reality exergame training. (**a**): Experimental intervention—participant using virtual reality exergame device. (**b**): Experimental intervention—home-based virtual reality exergame.

The intervention regimen for stroke included aerobic and resistance exercises, along with functional training encompassing the upper limb, trunk, lower limb, and whole body. Additionally, specific programs were created that considered the objectives and the exercise frequency, intensity, time, duration, and type, following the American College of Sports Medicine guidelines. The program for the intervention group will be the Nintendo Switch RingFit Adventure (RFA) (Figure 2). RFA is an exergame for the Nintendo Switch that utilizes virtual reality technology.

For this activity, the participant will need a Nintendo Switch console, a Ring-Con (a Pilates ring that the user holds), a wireless Joy-Con controller (one Joy-Con should be placed on the Ring-Con and the other should be attached to a leg strap on the player’s thigh), and a display screen. The program will be conducted twice a week for eight weeks. Each session will last 60 min, with 10 min each for warm-up and cool-down and 40 min for the main program. RFA is a fitness action role-playing game where the player’s physical movements drive the character’s actions on the screen, progressing the storyline through exercise. These movements are executed by engaging in physical activities using the Ring-Con and leg strap [41]. The Ring-Con is equipped with high-precision force and strain sensors, enabling it to detect and convert the participant’s actions, such as squeezing or stretching [42]. Additionally, the Joy-Con controller features a motion-infrared camera capable of monitoring the player’s heart rate [42]. RFA has the capability to estimate the optimal exercise intensity for each participant and adjust it based on physiological feedback, both lowering and increasing intensity levels [43]. Initially, the virtual coach sets the exercise intensity during the first game, and this is gradually fine-tuned based on subsequent game instructions. The study team will continuously monitor the experimental group participants, motivating them to complete the eight-week intervention, which will be conducted at 65–80% of each individual’s maximum heart rate measured at baseline. Participants will be required to exercise at a level between 65% and 80% of their maximum heart rate, as measured at the start of the study. They will be instructed to maintain an intensity level of between 12 and 16 on the rate of perceived exertion (RPE) scale [44], which is equivalent to “somewhat hard” to “hard”. On the other hand, the control group will continue their regular routine without any intervention during the study period.

### 2.7. Outcomes

All participants will undergo a baseline assessment and an assessment after 16 sessions, using the outcome measures listed in Table 2. We will collect sociodemographic and descriptive data through an ad hoc questionnaire. Primary outcomes will assess muscle strength (isokinetic muscle strength and grip strength), cardiopulmonary endurance (VO2peak and 6 min walking test), body composition (whole-body dual-energy X-ray absorptiometry and bioimpedance analysis), physical performance (short physical performance battery), static balance (Berg balance scale), dynamic balance and gait (Timed Up and Go test and 10 m walk test) to assess physical fitness. Secondary outcomes will assess the quality of life (Euro Quality of Life 5 Dimensions) and ability to perform activities of daily living (Barthel index).

#### 2.7.1. Muscular Strength

The isokinetic muscle test (using the Humac Norm system by CSMi in Stoughton, MA, USA) will assess the maximum muscle strength (peak torque) of the quadriceps. Isokinetic dynamometry is known for its objective and reliable nature when measuring maximal strength in stroke patients [45]. Participants will receive a comprehensive explanation of the isokinetic testing procedure to ensure they achieve optimal orientation. For the quadriceps evaluation test, participants will be seated with their seat back adjusted to a 70 degree angle from the horizontal plane. The motion axis of the knee joint will be determined by a transverse line through the femoral condyles. The dynamometer’s effort arm will be set based on the length of the crus, and waist belts and shoulder supports will be used to stabilize the trunk during each action, which will consist of 5 maximal contractions at an angular velocity of 60°/s. 

To assess upper extremity muscle strength, all participants will be measured using the hand dynamometer (TKK-5401; Takei Scientific Instruments, Tokyo, Japan) while sitting with their ipsilateral shoulder slightly adducted and at neutral rotation, elbow flexion at 90°, and forearm in a neutral position, with the wrist in a neutral position for assessing grip strength [46]. Each hand’s measurements will be taken two times, and the average value will be utilized for analysis.

#### 2.7.2. Aerobic Capacity

In order to evaluate aerobic capacity, we will opt for the measurement of the peak oxygen uptake. We will determine peak aerobic capacity by employing a maximal progressive cycle ergometer test [47]. Following a 3 min warm-up, the load will increment every 60 s in 10 W increments until the point of exhaustion or when the participant experiences discomfort or pain [48]. The initial load and progression will be tailored to each individual to ensure exhaustion is reached within 5–10 min. Expired air will be collected through a nose and mouth facemask, and an online respiratory gas exchange analyzer (K5 system, Cosmed, Rome, Italy) will record oxygen uptake and carbon dioxide release every 10 s. The peak oxygen uptake rate will be defined as the highest value of oxygen consumption recorded during any continuous 30 s interval of the cycle ergometer test. Heart rate will be continuously monitored throughout the test using a heart rate monitor, and two staff will be present at all times to oversee the procedure.

#### 2.7.3. Body Composition

We will use whole-body dual-energy X-ray absorptiometry (DEXA) with Discovery Wi (Hologic, Waltham, MA, USA) and bioelectrical impedance analysis (BIA) with InBody S10 (Inbody, Seoul, Republic of Korea) to examine body composition. DEXA will provide detailed information about both total and segmented fat-free lean and fat masses, while BIA will collect data on a variety of body composition metrics, including body mass index, body fat percentage, skeletal muscle mass, body weight, and waist-to-hip ratio. To measure participants’ height and weight, we will employ a stadiometer (BSM370; Inbody, Seoul, Republic of Korea).

#### 2.7.4. Functional Capacity

The Short Physical Performance Battery (SPPB) will examine three distinct functional aspects: balance, gait, and leg strength. The balance evaluation will involve maintaining balance for 10 s in three different foot positions: side-by-side, semi-tandem, and tandem [49]. The gait speed assessment will measure how fast one can walk during a 4 m walk test. The leg strength evaluation will measure the time it takes to complete five sit-to-stand repetitions. Each of these components will be rated on a scale from 0 to 4 points, with a maximum total score of 12. The SPPB’s remarkable sensitivity, as demonstrated by its cutoff score of ≤8 points, will suggest its potential as a valuable tool for screening sarcopenia in clinical settings. The SPPB will also show high reliability with an intraclass correlation coefficient (ICC) of 0.92.

#### 2.7.5. Balance

We will use the Berg balance scale (BBS), a dependable and valid assessment tool, to evaluate the static balance of individuals who have had a stroke [50]. The test comprises 14 tasks of varying difficulty, with each task rated on a scale from 0 (impossible) to 4 (completely independent performance), resulting in a maximum score of 56 points [50]. If a participant scores 45 points or lower, it will indicate poor balance and an increased risk of falling. Participants will have the option to utilize assistive devices and orthotics during the balance tests, and any such usage will be documented at the baseline to maintain consistent testing conditions throughout the exercise period.

We will utilize the Timed Up and Go (TUG) test to evaluate the dynamic balance of stroke survivors [51]. We have selected this test due to its straightforward and efficient assessment of functional mobility. The TUG will be applied to assess functional mobility and dynamic balance by measuring the time it takes for an individual to stand up from a chair, walk a distance of three meters, pivot, return to the chair, and sit down [51]. During the examination, participants will be expected to wear their regular footwear and make use of any mobility assistive devices they typically use. 

#### 2.7.6. Mobility

We will assess the maximal gait speed by having participants cover a 10 m distance on a 14 m course, providing them with a start. The participants will be directed to walk as quickly as possible by the staff and will complete two walking trials. They will be allowed to rest as needed between each trial, and the highest speed achieved in the best trials will be documented as the maximal gait speed. The 10 m walking test is a commonly used evaluation tool for a variety of patient groups and has a proven track record of reliability and validity in assessing the walking capability of individuals who have experienced a stroke [52].

#### 2.7.7. Secondary Outcomes

The Euro-Quality of Life 5 Dimensions (EQ-5D) comprises five items, each addressing a dimension of Health-Related Quality of Life (HRQoL), which include mobility, self-care, usual activities, pain or discomfort, and anxiety or depression [53]. Each of these items will be rated on a five-point scale, with options for “no problems”, “minor issues”, “moderate challenges”, “significant problems”, and “severe problems/incapable”.

The Barthel index (BI), which consists of 10 items designed to evaluate one’s capacity for everyday activities, is a common tool in clinical practice. While some researchers have made adjustments to the original Barthel scale, Quinn et al. strongly advocate for the adoption of a single, consistent version of the BI scale for stroke patients. They specifically endorse the use of the 10 item scale with a total score that spans from 0 to 100, with increments of 5 points, as the established BI standard for stroke assessment [54].

### 2.8. Participant Timeline

The participant timeline shows the timeline for recruiting, experimenting, and evaluating study participants according to the SPIRIT statement in Appendix A [40].

### 2.9. Data Collection Methods

All the measures to assess outcomes will be gathered both before and after the intervention across the 16 sessions, with the exception of the descriptive and sociodemographic variables. 

### 2.10. Statistical Analysis

The sample size is determined based on a prior study [55]. We will perform a power analysis using G*Power software version 3.1.2 [56], employing an effect size index of 0.5 for all outcome measures, a significance level of 0.05, and a minimum type II error (80% power) for all outcome measures. To reach the intended sample size of 29 participants per group, we will recruit 30 participants from each of the four groups, resulting in a total of 120 participants.

To evaluate the comparability between the experimental and control groups, we will assess participant characteristics and baseline comparisons. We will provide a table illustrating the attributes of participants allocated to each group. Discrete variables will be expressed as frequencies and percentages, while continuous variables and time intervals will be summarized with means, standard deviations, medians, and interquartile ranges. This ensures that both groups are similar at the outset.

Our statistical analyses will follow an intention-to-treat approach. For primary outcomes, we will begin by assessing the normality of the dependent variables using the Kolmogorov–Smirnov test. Depending on the distribution, we will describe the dependent variables using either means and standard deviations or medians and interquartile ranges. To address the primary objective, we will perform hypothesis tests for each primary outcome, with the alternative hypothesis suggesting that outcomes in the experimental group will be superior to those in the control group. Additionally, we will provide 95% confidence intervals for all estimates. For independent samples with a normal distribution, we will employ the Student’s *t*-test, and for non-normally distributed data, we will use the Mann–Whitney U test.

In the analysis of secondary outcomes, we will compare hypotheses related to quality of life and provide estimates with 95% confidence intervals. Furthermore, we will construct a regression model to explore potential correlations between quality of life and factors such as muscle strength, cardiopulmonary endurance, flexibility, functional capacity, static balance, dynamic balance, and gait. We will assess the strength of these relationships using an analysis of variance F-test and individual t-tests, presenting the results as odds ratios and their 95% confidence intervals.

## 3. Discussion

This multicenter randomized controlled trial will be the first study to determine the effectiveness of home-based VRET in post-stroke patients discharged from the hospital with a doctor’s note in South Korea. Best practice in stroke care advocates providing care in patients’ homes or communities based on their needs and preferences [57]. Home-based rehabilitative exercise can be provided using VRET, a new modality [13]. VRET systems are compact, user-friendly, and can be remotely monitored by clinicians, making them ideal for home use [13]. Home-based VRET is suitable for patients after mild stroke who have been discharged from acute care in a hospital and wish to recover their normal physical function. After discharge from inpatient rehabilitation, it is advantageous for patients who have had severe stroke to maintain or increase the intensity of their treatment. VRET is a supplementary therapy to traditional in-person rehabilitation, which usually occurs once to thrice per week. Continuing therapeutic exercise after discharge from formal rehabilitation is possible using this approach. A previous study has shown that increasing rehabilitative exercise by 15 h or more is necessary to enhance post-stroke function [58]. Patients with stroke may find it easier to achieve the required amount of exercise if they have the flexibility to perform daily exercises at home on their schedules.

Home-based VRET has the potential to increase exercise intensity, promote recovery, and improve physical fitness. This study is the first step toward testing this hypothesis. Currently, only one small randomized controlled trial has been conducted on VRT in a home setting. A previous study used a single Kinect-based activity performed thrice a week at home in addition to two clinic visits per week [27]. Our study will involve a VRET system with a wide range of exercises, allowing for greater customization to meet the treatment goals of patients with stroke. In addition, the VRET intervention in our protocol will be entirely home-based.

We anticipate that VRET will be considered feasible because the equipment can be conveniently installed in participants’ homes, and the participants can learn and progress effectively through VRET. Furthermore, we anticipate that participants will enjoy VRT and recognize its benefits in their recovery process without experiencing adverse effects such as injuries or falls.

The study findings will provide a means for post-stroke patients to safely engage in VRET and home-based exercise following their hospital discharge. We intend to publish our findings in journals and conferences and to disseminate them through conferences and meetings related to stroke.

It is important to note that our study will be constrained by a small sample size, consisting of just 15 participants in each team within the experimental group. While conducting a feasibility study is a prudent step before committing resources to a full research project, this limited sample size might potentially hinder our ability to detect significant differences between the groups.

After conducting these trials, our research team plans to conduct a definitive study to test the effectiveness of home-based VRET in improving physical outcomes, such as cardiopulmonary endurance, muscular strength, functional capacities, gait, and overall function. The results will provide information for future randomized controlled trials regarding parameters, including primary and secondary outcome measures, number of weeks of VRET, length of VRET sessions, frequency per week, sample size, and control intervention. It is important to have practical insights regarding the connections to home-based VRET after discharge from the hospital, which will be instrumental in shaping forthcoming research initiatives. We are exploring how doctors and exercise instructors can collaborate to support the use of VRET for stroke patients post-hospitalization. Our aim is to further develop this field in the times ahead. Notably, there is a rising inclination toward the use of technology in home-based settings. In this regard, VRET is anticipated to boost rehabilitation intensity and contribute to improved physical outcomes for individuals recovering from stroke.

## 4. Conclusions

Our research has the potential to offer fresh perspectives on the efficiency of VRET programs for post-stroke patients, enabling them to engage in home-based VRET safely with a physician’s authorization following hospital discharge. We also aim to gain insights into the feasibility of home-based exercise for post-stroke patients after leaving the hospital. Furthermore, if the novel exercise training method proves to be effective, it could yield valuable data for the formulation of more comprehensive protocols for stroke patients in the future.

## Figures and Tables

**Table 1:** Intervention protocol.

Warm-Up/Cool-Down		Upper Limb	Trunk	Lower Limb	Whole Body
Aerobic	Flexibility				
Walking	Static/ dynamic stretching	- Back press - Overhead press - Front press - Bow pull - Shoulder press - Triceps kickback - Overhead arm twist - Overhead arm spin	- Overhead side bend - Pendulum bend - Overhead bend - Knee-to-chest - Seated forward press - Plank - Leg raise - Standing twist - Russian twist - Flutter kick - Seated ring raise - Mountain climber - Leg scissors - Fan pose - Boat pose - Standing forward fold - Open and close Leg raise	- Squat - Wide squat - Overhead squat - Thigh press - Hip lift - Chair pose	- Overhead lunge twist - Overhead hip shake - Knee lift - Side step - Ring raise combo - Knee-lift combo - Tree pose - Hinge pose - Revolved crescent Lunge pose - Warrior I pose - Warrior II pose - Warrior III pose

**Table 2:** Data collection: medical part and anthropometric part data.

Medical Part	Age	Years
	Gender	Male or female
	Stroke Type	Ischemic or Hemorrhagic
	Hypertension, anemia, dyspnea or asthma, orthostatic hypotension, diabetes, medications for heart disease, coronary stent, epilepsy, medications for anticoagulants, medications for depression, acute low back pain within 4 weeks, walking due to joint pain, medications for osteoporosis, spine, hip, or femur fractures due to osteoporosis, hip or femur fractures due to falls	Yes or no
Anthropometric Part	Blood pressure	mmHg
	Height	cm
	Weight	kg
	Body mass index	Underweight/normal weight/overweight
	Days of discharge from the hospital	Number of days

## Data Availability

The data will be made available by the authors on reasonable request.

## Appendix A

**Table A1:** Study Period: Schedule of Enrollment, Interventions, and Assessments of This Study.

Study Period				
	Enrollment	Allocation	Post-Allocation	Closeout
Timepoint, baseline	t	0	T16	tx
Enrollment				
Eligibility screening	X			
Informed consent	X			
(Participant) Self-report questionnaires	X			
(Clinicians) Confirm participants’ self-report questionnaires and medical history and write doctor’s notes	X			
Allocation		X		
Assessments				
Baseline variables		X		
Post-intervention variables			X	X
Intervention				
Experimental group			X	X
